# Optimizing the Use of RTKLIB for Smartphone-Based GNSS Measurements

**DOI:** 10.3390/s22103825

**Published:** 2022-05-18

**Authors:** Tim Everett, Trey Taylor, Dong-Kyeong Lee, Dennis M. Akos

**Affiliations:** 1 RTK Consultants LLC, Niwot, CO 80503, USA; tim.everett3@gmail.com; 2 Aerospace Engineering Sciences, University of Colorado Boulder, Boulder, CO 80309, USA; fred.tayloriii@colorado.edu (T.T.); dma@colorado.edu (D.M.A.)

**Keywords:** Android, smartphone, RTK, PPK, GNSS, RTKLIB, decimeter, Google, carrier, DGNSS

## Abstract

The Google Smartphone Decimeter Challenge (GSDC) was a competition held in 2021, where data from a variety of instruments useful for determining a phone’s position (signals from GPS satellites, accelerometer readings, gyroscope readings, etc.) using Android smartphones were provided to be processed/assessed in regard to the most accurate determination of the longitude and latitude of user positions. One of the tools that can be utilized to process the GNSS measurements is RTKLIB. RTKLIB is an open-source GNSS processing software tool that can be used with the GNSS measurements, including code, carrier, and doppler measurements, to provide real-time kinematic (RTK), precise point positioning (PPP), and post-processed kinematic (PPK) solutions. In the GSDC, we focused on the PPK capabilities of RTKLIB, as the challenge only required post-processing of past data. Although PPK positioning is expected to provide sub-meter level accuracies, the lower quality of the Android measurements compared to geodetic receivers makes this performance difficult to achieve consistently. Another latent issue is that the original RTKLIB created by Tomoji Takasu is aimed at commercial GNSS receivers rather than smartphones. Therefore, the performance of the original RTKLIB for the GSDC is limited. Consequently, adjustments to both the code-base and the default settings are suggested. When implemented, these changes allowed RTKLIB processing to score 5th place, based on the performance submissions of the prior GSDC competition. Detailed information on what was changed, and the steps to replicate the final results, are presented in the paper. Moreover, the updated code-base, with all the implemented changes, is provided in the public repository. This paper outlines a procedure to optimize the use of RTKLIB for Android smartphone measurements, highlighting the changes needed given the low-quality measurements from the mobile phone platform (relative to the survey grade GNSS receiver), which can be used as a basis point for further optimization for future GSDC competitions.

## 1. Introduction

### 1.1. Real-Time Kinematic (RTK)

Multiple global navigation satellite system (GNSS) measurements exist that can be used for position, velocity, and time (PVT) computations. These include code, carrier, and doppler measurements. RTK uses carrier phase measurements to provide more precise PVT than code-based positioning. Theoretically, RTK is able to provide centimeter-level positioning to its users [1]. Multiple GNSS receivers exist that provide RTK solutions, but commercial software packages are expensive [2]. A list of the available open-source and closed-source GNSS packages with RTK capabilities is provided by the National Geodetic Survey (NGS) [3]. RTKLIB is one of the open-source packages available to the public.

### 1.2. What Is RTKLIB?

RTKLIB was first developed in April 2006 by Tomoji Takasu, first released to the public as an open source in January 2009, and is currently distributed under a BSD 2-clause license [4]. The latest version from Takasu’s branch is 2.4.3 b34, which was released on 29 December 2020, but multiple forks exist, including the one from the author of this paper, Tim Everett. For user interface, RTKLIB offers graphic user interface (GUI) access points (AP) on Windows and Console apps on both Windows and Linux environments. RTKLIB is a powerful GNSS data analysis tool that can process various types of GNSS, data including receiver independent exchange format (RINEX) [5] and radio technical commission for maritime services (RTCM) formats [6]. Using the input data, it is able to carry out various position computations, including single point positioning (SPP), differential GNSS (DGNSS), real-time kinematic GNSS (RTK-GNSS), post-processing kinematic GNSS (PPK-GNSS), and precise point positioning (PPP). Over the years, numerous updates have been made to the RTKLIB for the support of multi-constellation and multi-frequencies. For this paper, we look at the PPK solution, since it does not require any real-time navigation solution computations, and PPK is able to provide higher accuracy position solutions than SPP and PPP [7]. Furthermore, all the GNSS observables and ephemeris information from nearby base stations required for PPK are available from various public servers.

### 1.3. Google Smartphone Decimeter Challenge (GSDC)

GSDC was a Kaggle competition held by Google in 2021 (with a second expected offering in 2022). The objective was to generate the most accurate position solutions for a large number of raw observation data sets collected using Android devices inside a moving vehicle in the San Francisco Bay area [8]. In 2021, two sets of data, training and test, were provided by Google on Kaggle [9]. There were 29 routes and 73 smartphone data in the train set, and 19 routes and 45 data in the test set. There were multiple data for each route because multiple Android devices, spaced approximately 20 cm apart, were logging the data at the same time. During the drive, truth data was also collected using a NovAtel SPAN ISA-100C unit. This is because the SPAN unit is able to provide a horizontal accuracy of up to 0.01 m RMS [10]. As the smartphones were not in the same location as the antenna, their relative displacements with respect to the NovAtel antenna were taken into account for the smartphone truth determination. The training set was provided with the corresponding truth positions, but the truth for the test set was used for the Kaggle leaderboard determination. The data collected was provided in both RINEX and raw GnssLogger format. Although the raw format is what is logged by the smartphone, Google also provided a translation of the raw measurements into the RINEX format for those who preferred the more commonly used format. The measurements were filtered by Google during the conversion, and more details on this are provided in the Data Conversion section.

The drive routes can be classified into highway, street, and downtown, depending on the amount of expected multipath in the signals [11]. The highway is mostly open-sky, the street is open-sky with some attenuation from trees and nearby buildings, and downtown is heavily affected by attenuation and multipath from high-rise buildings. For the phone setup, the devices were located on the dashboard of the vehicles with no ground plane, which means that they were exposed to significant amounts of additional signal attenuation, and multipath at least at the level as to what would be expected if the antennas were placed on the roof of the vehicle.

The scores for the competition were determined by averaging the 50th percentile and 95th percentile errors of the computed latitude and longitude positions at each time epoch. The errors were computed using truth provided by the NovAtel SPAN unit. As the truth was not released for the test set, the scores were available upon submitting a list of the computed positions to the Kaggle website. In order to allow the participants to test the Kaggle website interface, a baseline results file was provided by Google as well. The algorithms used by Google to obtain the positions in the baseline file were proprietary, but some details about how the results were processed were provided to all participants [12].

### 1.4. Performance of RTKLIB and GSDC Participants

Coupled with commercial receivers such as u-blox, RTKLIB has been able to provide centimeter level of accuracy [13]. However, as RTKLIB was originally designed for commercial GNSS receivers with survey type measurements, multiple adaptations are required for its use in GSDC, where only the GNSS measurements from smartphones are available. The smartphones used in GSDC (Google Pixel4 variants, Samsung S series variants, and Xiaomi Mi8) have several differences from commercial GNSS receivers, particularly with respect to antenna design/performance [14]. Consequently, the quality of the measurements is worse, leading to greater signal noise, increased number of carrier phase cycle slips, and increased multipath. This issue is illustrated by Qiong [15], when raw GNSS measurements from a Xiaomi Mi8 smartphone and a geodetic receiver collected at the same time were processed using RTKLIB. When GPS L1 measurements from both devices were processed using the same kinematic PPP mode, the geodetic receiver had horizontal position errors of approximately 1 m, while the smartphone’s errors were approximately 3–5 m, due to fewer observed satellites and lower carrier to noise ratio density (C/N_0_) for the tracked satellites.

In the 2021 GSDC, the 1st-place winner used factor graph optimization, coupled with Takasu’s version of the RTKLIB, to obtain a score of 1.62 m [11]. Other teams used Google’s baseline Android navigation engine [16] or their own proprietary navigation engines [17]. In the Results section of the paper, the performance of the updated RTKLIB against other navigation engines will be provided using the official Kaggle scoring system. The comparison candidates are Google’s baseline solution and the scores of other participants. The goal will be to assess where the performance of the suggested RTKLIB adaptation stands among other available navigation engines used in the competition.

## 2. Strategies

### 2.1. PPK Solution

Post-processed kinematics, or PPK, is a popular use case for RTKLIB, as it allows for high precision position results for previously recorded kinematic data. The PPK solution can utilize the provided GNSS observation file, an additional observation file from a nearby base station, and multi-constellation ephemeris files in order to perform precise positioning at each epoch. In addition to pseudorange measurements, PPK also utilizes the available carrier phase in the navigation solution. PPK in RTKLIB also allows the user to implement a noncausal Kalman filter computation, operating both forwards and backwards in time. By doing this, RTKLIB is able to detect directional anomalies, such as cycle slips, and combine both the results to produce results with better accuracy and integrity.

### 2.2. Kinematic Solution Algorithm

The navigation engine of RTKLIB is based on extended Kalman filter (EKF) and double differencing with respect to a nearby base station. Although the different modes and algorithms implemented for the RTKLIB are available in the user manual [18], a summary of the relevant algorithms utilized for GSDC is provided below. Moreover, although the manual explains the algorithms for estimating the position and velocity states of the receiver, the latest RTKLIB also includes the added acceleration states as well, so the equations below address this update. The EKF is used to compute the estimated state vector ${\hat{x}}_{k}$, and its covariance matrix $P_{k}$, for epoch $t_{k}$, using a measurement vector $y_{k}$.

(1) $${\hat{x}}_{k}^{+}={\hat{x}}_{k}^{-}+K_{k}(y_{k}-h\left({\hat{x}}_{k}^{-}\right)$$

(2) $$P_{k}^{+}=\left(I-K_{k}H\left({\hat{x}}_{k}^{-}\right)\right)P_{k}^{-}$$

(3) $$P_{k}^{+}=\left(I-K_{k}H\left({\hat{x}}_{k}^{-}\right)\right)P_{k}^{-}$$

(4) $${\hat{x}}_{k+1}^{-}=F_{k}^{k+1}{\hat{x}}_{k}^{+}$$

(5) $$P_{k+1}^{-}=F_{k}^{k+1}P_{k}^{+}F_{k}^{k+1}{}^{T}+Q_{k}^{k+1}$$

where the +/− signs indicate whether it is before or after the measurement update. F and Q are the state transition matrix and the system noise covariance matrix, respectively, and m is the number of satellites observed and used in the computation. For a given receiver r, stationary base station b, and satellites j and k, we can take the double difference of the pseudorange P, and carrier phase measurements ϕ.

(6) $$P_{rb,i}^{jk}=\rho_{rb}^{jk}+\epsilon_{P}$$

(7) $$\Phi_{rb,i}^{jk}=\rho_{rb}^{jk}+\lambda \left(B_{rb,i}^{j}-B_{rb,i}^{k}\right)+\epsilon_{\phi }$$

where ρ is the geometric range, ϵ is the noise, λ and i are the wavelength and corresponding index of the GNSS signal which is, in this case, L1 and L5, and B is the single differenced integer ambiguity. The double difference effectively removes the atmospheric error effects from the measurements. The state vector x we are solving for would include the position $r_{r}$, velocity $v_{r}$, and acceleration $a_{r}$ of the receiver.

(8) $$x=\left[\begin{array}{c} r_{r}\\ v_{r}\\ a_{r}\\ B_{1}\\ B_{5} \end{array}\right]\;B_{i}=\left[\begin{array}{c} B_{rb,i}^{1}\\ B_{rb,i}^{2}\\ \ldots \\ B_{rb,i}^{m} \end{array}\right]$$

The measurement vector y is defined with respect to a single satellite with the highest elevation indexed as 1. Although this is effective for a high elevation satellite in open-sky conditions, it is important to note that if this satellite has anomalies, this could negatively affect the quality of the final solution.

(9) $$y=\left[\begin{array}{c} \phi_{1}\\ \phi_{5}\\ P_{1}\\ P_{5} \end{array}\right]\;\phi_{i}=\left[\begin{array}{c} \phi_{rb,i}^{12}\\ \phi_{rb,i}^{13}\\ \ldots \\ \phi_{rb,i}^{1m} \end{array}\right]\;P_{i}=\left[\begin{array}{c} P_{rb,i}^{12}\\ P_{rb,i}^{13}\\ \ldots \\ P_{rb,i}^{1m} \end{array}\right]$$

For the measurement update, we write the measurement model vector h(x), the matrix of partial derivatives H(x), and the covariance matrix of measurement errors R, as follows:

(10) $$h\left(x\right)=\left[\begin{array}{c} h_{\phi ,1}\\ h_{\phi ,5}\\ h_{P,1}\\ h_{P,5} \end{array}\right]\;H\left(x\right)=\left[\begin{array}{cc} \begin{array}{cc} -DE & 0\\ -DE & 0 \end{array} & \begin{array}{cc} \lambda_{1}D & 0\\ 0 & \lambda_{5}D \end{array}\\ \begin{array}{cc} -DE & 0\\ -DE & 0 \end{array} & \begin{array}{cc} 0 & 0\\ 0 & 0 \end{array} \end{array}\right]$$

(11) $$R=diag\left(DR_{\phi ,1}D^{T},DR_{\phi ,5}D^{T},DR_{P,1}D^{T},DR_{P,5}D^{T}\right)\;$$

(12) $$h_{\phi ,i}=\left[\begin{array}{c} \rho_{rb}^{12}+\lambda_{i}\left(B_{rb}^{1}-B_{rb}^{2}\right)\\ \rho_{rb}^{13}+\lambda_{i}\left(B_{rb}^{1}-B_{rb}^{3}\right)\\ \ldots \\ \rho_{rb}^{1m}+\lambda_{i}\left(B_{rb}^{1}-B_{rb}^{m}\right) \end{array}\right]h_{P,i}=\left[\begin{array}{c} \rho_{rb}^{12}\\ \rho_{rb}^{13}\\ \ldots \\ \rho_{rb}^{1m} \end{array}\right]\;$$

(13) $$D=\left[\begin{array}{ccc} \begin{array}{ccc} 1 & -1 & 0\\ 1 & 0 & -1 \end{array} & \cdots & \begin{array}{c} 0\\ 0 \end{array}\\ \vdots & \ddots & \vdots \\ \begin{array}{ccc} 1 & 0 & 0 \end{array} & \cdots & -1 \end{array}\right]\;E={\left[e_{r}^{1},e_{r}^{2},\ldots ,e_{r}^{m}\right]}^{T}$$

(14) $$R_{\phi ,i}=diag\left(2\sigma_{\phi ,i}^{1}{}^{2},2\sigma_{\phi ,i}^{2}{}^{2},\ldots ,2\sigma_{\phi ,i}^{m}{}^{2}\right)R_{P,i}=diag\left(2\sigma_{P,i}^{1}{}^{2},2\sigma_{P,i}^{2}{}^{2},\ldots ,2\sigma_{P,i}^{m}{}^{2}\right)\;$$

where e is the light of sight (LOS) vector between the satellite and the receiver, σ is the standard deviation of the pseudorange and carrier phase measurement error for each satellite. For the time update of the EKF, the positioning mode is kinematic and the receiver dynamics are turned on. Therefore, the state transition matrix and the system noise covariance matrix are defined as below.

(15) $$F_{k}^{k+1}=\left[\begin{array}{cc} \begin{array}{ccc} I_{3\times 3} & I_{3\times 3}\tau_{r} & I_{3\times 3}\frac{\tau_{r}^{2}}{2}\\ 0 & I_{3\times 3} & I_{3\times 3}\tau_{r}\\ 0 & 0 & I_{3\times 3} \end{array} & \;\\ \; & I_{\left(2m-2\right)\times \left(2m-2\right)} \end{array}\right]$$

(16) $$Q_{k}^{k+1}=diag\left(0_{3\times 3},0_{3\times 3},Q_{a},0_{\left(2m-2\right)\times \left(2m-2\right)}\right)$$

(17) $$Q_{a}=E_{r}^{T}\mathrm{diag}\left(\sigma_{ae}^{2}\tau_{r},\sigma_{an}^{2}\tau_{r},\sigma_{au}^{2}\tau_{r}\right)E_{r}$$

(18) $$\tau_{r}=t_{k+1}-t_{k}\;$$

where $\tau_{r}$ is the receiver sampling interval in seconds between epochs k and k+1, and $\left(\sigma_{ae},\sigma_{an},\sigma_{au}\right)$ are the east, north, and up components of the receiver acceleration system process noise.

### 2.3. Satellite and Correction Data Selection

In order to obtain PPK solutions, observation files from nearby base stations and satellite ephemeris files are required. The ephemeris files were pulled from the International GNSS Service (IGS) website. The files chosen were BRDM files that contain navigation parameters for all GNSS constellations over the course of the data collection day. The BRDM files are generated as part of the Multi-GNSS experiment (MGEX), where the signals from multiple constellations, including GPS, GLONASS, BeiDou, Galileo, QZSS, NAVIC, and SBAS are merged into combined broadcast ephemeris files [19]. The base station observation files were pulled from the National Oceanic and Atmospheric Administration (NOAA) National Geodetic Survey (NGS) website for the Stanford Linear Accelerator Center (SLAC) station. SLAC, currently known as the National Accelerator Laboratory, is one of the 17 Department of Energy national laboratories, and is located at an approximate distance of at most 35 km from the route, which is within the maximum recommended range of 50 km for decimeter-level accuracy RTK or PPK [7]. The provision of GPS, GLONASS, and Galileo observation data, along with the SLAC station’s relative proximity to the data collection routes, made it a desirable site to utilize when attempting to eliminate atmospheric and clock errors in the PPK solution.

### 2.4. Data Conversion

The competition data provided by Google included both raw GNSSLogger and RINEX formats. The RINEX format is compatible with RTKLIB, but the method and parameters used to convert the raw phone data to RINEX was not fully provided to the challenge participants and did not appear to fully align with methodologies suggested by Google in the competition discussion pages. As a result, it was beneficial to convert the raw phone data to RINEX using available open-source tools, as opposed to utilizing the pre-processed files, to provide more user control over the pre-processing data conversion process. To accomplish this conversion, the Rokubun Android GnssLogger to RINEX converter python code was chosen as a starting point [20]. This code is split into two sections, gnsslogger.py, which pulls out and organizes phone data, and rinex3.py that takes in the organized data and writes it out in the RINEX version 3 format. Within both sets of code, adjustments were made to improve the resulting RINEX files. The details of the adjustments and improvements are documented below.

In gnsslogger.py, changes were made to expand compatibility to all phone types used in the competition and to more closely follow filtering rules described by Google in a competition discussion post describing their methodology for generating the included baseline solutions [12]. These changes were primarily integrated by including an observation filter function that served to reject invalid or very low-quality observations as defined by:

- C/N_0_ was less than 20 dB-Hz;
- Received space vehicle time uncertainty was greater than 500 nanoseconds;
- Maximum pseudorange uncertainty was greater than 150 m;
- Multipath indicator was set greater than 0;
- Maximum carrier phase uncertainty was greater than 0.1 m;
- Status of the code lock was invalid;
- TOW or TOD values were not decoded and set for all constellations;
- Constellation identifier was invalid.

Finally, the script was modified to provide an option to ignore the cycle slip and half-cycle ambiguity flags that were present in the provided raw data files, as investigating these components showed that their inclusion resulted in usable data often being thrown out. This option was enabled for the processing of the raw data for this solution.

The changes to the rinex.py file were much less involved. The core change made to this script was to replace the single character, unused legacy signal to noise ratio (SNR) field of the RINEX file with the pseudorange and carrier phase uncertainty estimates from the receiver. These receiver estimates can be included in RTKLIB’s determination of measurement weights, but for this solution, only elevation-based weighting was utilized.

### 2.5. Code Changes to the RTKLIB Demo5

In order to get high levels of performance from the RTKLIB PPK processing, changes were made to the demo5 b34e version of RTKLIB. The demo5 code is an open-source, publicly available fork from the RTKLIB 2.4.3 code, and it is maintained on GitHub [21]. It is focused on improving solution performance, reliability, and robustness, particularly for low-cost single and dual frequency receivers. It is kept closely synchronized to the 2.4.3 code version, and all updates from that code are generally ported into the demo5 code. The vast majority of the code is common between the two versions, but some of the more significant differences between them are in the ambiguity resolution algorithms, particularly in the approach to partial ambiguity resolution and the options for dealing with the GLONASS inter-channel biases. However, it should be noted that ambiguity resolution was disabled for this solution, so the differences between the two versions of code are less significant in this case.

The changes made to the demo5 b34e code specifically for this experiment fell into one of two categories. The first and most important is the approach to cycle slip detection. The unmodified RTKLIB code has three methods for detecting cycle slips. The cycle slips can be flagged by the receiver, they can be detected as a Kalman filter error larger than a specified “outlier” threshold, or if dual frequency measurements are available, by using geometry-free linear combinations to detect phase jumps. Cycle slips in the smartphone observations are much more frequent than is typical from higher quality receivers. In addition, the reliability of the receiver cycle-slip flags appears to be much lower, both for false-positives and for false-negatives. In order to compensate for the large number of cycle slips and the low confidence in the receiver to accurately flag them, it was required to improve RTKLIB’s ability to detect the cycle slips through other means. The method used in this solution leveraged changing the demo5 code to enable the ability to detect cycle slips using doppler measurements. By checking the difference between the doppler measurement and the change in carrier phase measurement, as carrier phase is essentially the accumulated doppler range, the cycle slips can be detected. This method is included in the open-source versions, but is commented out due to a clock jump issue. By making changes to the code that allow it to process all satellites at an epoch in one call as opposed to processing all of them individually, the common-mode effect of the clock jump can be minimized. If there is a jump in all satellite channels, this would be classified as a clock discontinuity, and if it is present in a subset of channels, it would be identified as a cycle slip.

Other changes were made to reduce the number of observations that were unnecessarily discarded, allowing the solution to degrade more gracefully for poor data sets. An example of this was the adjustment of a check for valid observation data. For this check, instead of discarding any data that contained only pseudorange measurements, the code kept this incomplete data. Due to the fact that some of the Google data could be poor and often included incomplete measurement sets that lacked the carrier, changing this observation check was able to prevent good pseudorange data from being discarded. Additionally, another check in the code that sought to identify valid epoch solutions by ensuring that all epochs had four or more valid carrier phase double differences was changed. Almost all the epochs had at least four valid pseudorange double differences, it was considered restrictive to simply reject these; instead, we chose to use the float solution derived from the pseudoranges when the epoch did not have four or more carrier phase double differences. Continuing with the topic of the double differencing, an adjustment was made that allowed the code to avoid using a satellite with a cycle slip as a reference satellite, as previously, the code just utilized the satellite with the highest elevation. Additionally, a bug was identified and fixed in the geometry-free cycle slip detection, where the code was not checking the L1 frequency for cycle slips and thus, was not progressing as it was supposed to, given a case where only L1 measurements encountered cycle slips.

### 2.6. Changes to RTKLIB Settings

In addition to the RTKLIB code changes, configuration settings can be adjusted to better align with the expected data input, as well as leverage the code changes made [22]. As a starting point, a config file was used from a previous experiment made with some less challenging smartphone observations [23]. This previous experiment worked with data collected by a Xiaomi Mi8 phone and includes additional changes from the f9p_ppk.conf file provided with the demo5 code not discussed in this section, as they are minor and were specifically tuned to that dataset. The important configuration settings used to produce the results presented will be broken down based on whether they were settings for the processing, adjustments to the outputs, or changes made to the statistics. A summary of the changes with respect to the default f9p_ppk.conf demo5 b34e code is provided in Table 1.

Several changes were made to better accommodate the Google data and perform some simple filtering. First, the positioning mode was set to kinematic, as that is the type of data Google provides for the challenge, and only the GPS, GLONASS, and Galileo constellations were selected for use, as the SLAC station observations used only contain these. Leveraging the ability of the code to use GPS L5 and Galileo E5a, these frequencies were enabled, along with the L1 and L2 frequencies. Settings that were retained from the previous smartphone experiment to filter out some of the lowest quality observations included a 15 degree elevation mask, and a 24 dB-Hz C/N_0_ filter. Although this level of C/N_0_ is low for decent carrier phase measurements, as RTKLIB does not support separate thresholds for carrier and code, this value was selected to preserve the acceptable code measurements. Furthermore, this threshold was not an assessment of absolute signal quality, but a means to exclude the tail of the distribution. In addition, this is a higher threshold compared to the 20 dB-Hz used by Google in their code-only positioning for the baseline solutions. The solution was run in combined no phase reset mode, which includes running the Kalman filter over the data in both the forward direction and the backwards direction, then combining the results without resetting the ambiguity estimates between the two directions. Continuing with more settings, first, the ambiguity resolution was disabled, as errors within the dataset were too large to resolve consistently, resulting in false fixes and degrading overall solution quality. With the doppler slip now enabled in the RTKLIB code, it could be used, along with geometry-free detection and outlier detection, to identify slips in the carrier. The geometry-free slip threshold was 0.05 m by default, but this was deemed too small to account for the larger errors present in the datasets, so the value was increased to 0.1 m. The doppler slip threshold was set to 5 Hz.

The final changes made to the RTKLIB configuration file revolved around the output and the statistics of those outputs. The output configuration showed no changes, other than to change the time format, which was switched to “ww ssss GPST”, which is much closer to the format Google expects in the submitted results. The statistical changes to the configuration file were made to increase the weighting given to the L5 pseudorange measurements, as well as to account for lower confidence in the carrier phase bias due to the increased possibility of missed slips. The L5 phase error ratio was reduced from 300 to 100 due to L5′s higher signal strength and longer codes relative to L1, which results in smaller pseudorange errors. The carrier-phase bias was set to 0.01 cycles. Some of these values were decided upon by trial-and-error, using a select number of datasets from the training set that showed improvement to the overall solution using the values defined above.

### 2.7. Data Merge

**Figure 1:**
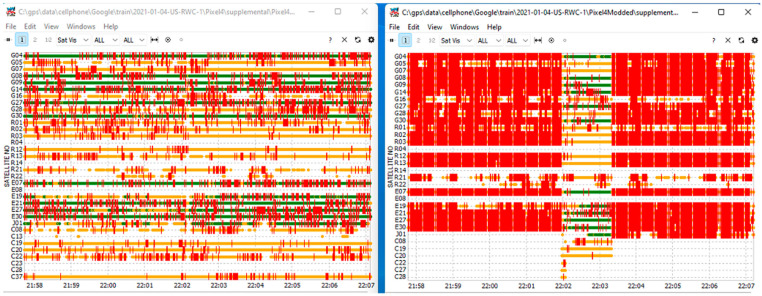
Availability visualization of all satellites present in the observation data for the Pixel 4 and Pixel 4 Modded. The left plot is for Pixel 4, and the right plot is for Pixel 4 Modded. The red markers indicate the flagged potential cycle slips present in the measurements.

Some of the phone data files were of particularly low quality and contained continuous cycle slips on all satellites for large portions of the data. The raw data for these files indicated hardware clock discontinuities for every epoch, which were potentially caused by the phone’s duty cycling the GNSS tracking during data collection. An example of this can be seen in Figure 1, which shows simultaneous observations taken from two adjacent phones, where the red ticks indicate the flagged cycle slips. With little to no valid carrier phase observations in the degraded files, these solutions tended to have very large position errors. Fortunately, the poor solutions also had very large error estimates, and there was at least one dataset in each collection drive that produced good results. Since the distance between the phones within the same collection scenario was small relative to the position errors, merging the solutions for datasets within a collection proved to be an effective method to minimize errors, not only in the problematic datasets, but also for all of the scenarios. The merge was carried out in the position domain instead of the measurement domain, because valid carrier phase measurements were available in at least one of the phones for each epoch, and the position domain merge was sufficient to leverage the benefits of carrier-enhanced positions. Furthermore, due to the approximate 20 cm physical separation between the phones, if the relative displacement vectors are not accounted for, a successful merge of the carrier phase measurements is difficult.

The merge was performed by combining the solutions output by RTKLIB for each data collection drive using a weighted approach. The weights were computed as the inverse of the variance of the data naturally computed by RTKLIB. If the weights were approximately zero, they were discarded and the remaining weights which passed this filter were utilized to compute a weighted average merged phone solution. This solution was then distributed back to the individual phones on the drive. The phone merge introduced a minimal error, as the phones were placed approximately 0.2 m from each other on the car dashboard [8], but the improvement to the overall solution that resulted from removing the poor phone data outweighed the small phone distance error.

## 3. Results

To gain an understanding of the performance of the current version of the RTKLIB with respect to Google’s baseline solutions, both the RTKLIB and baseline solutions for the training set were assessed using the provided truth data. Prior to the analysis, training datasets with clock discontinuities were removed, as clock discontinuities suggest no valid carrier phase measurements for that epoch, which will throw off the proposed RTKLIB algorithm. If we look at these problematic datasets, whenever there were discontinuities, the position errors exceeded 100 m. Although the discontinuities exist for the GSDC test set as well, the aforementioned data merge mitigates this issue. We perform the exclusion for the train set analysis to look at the accuracy of the RTKLIB solutions prior to the merge. The excluded data and their number of discontinuities is provided in Table 2.

**Figure 2:**
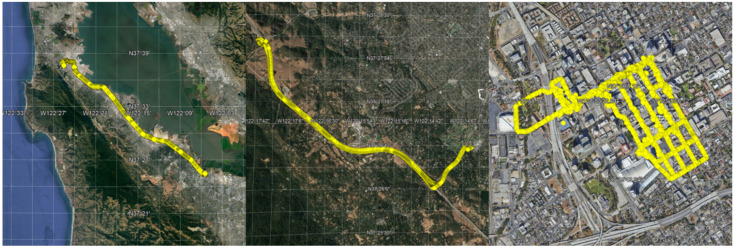
Illustration of the different scenarios: Highway (**left**), Street (**middle**), and Urban Canyon (**right**). Each scenario is representative of various GNSS environments: open-sky, mixed, and multipath.

After the data screening, the training data was separated into three scenarios: highway, street, and urban canyon. Highway indicates all the drives between San Francisco and San Jose, which are open-sky dominant. Urban canyon indicates the drives inside downtown San Jose, which are multipath dominant. Street indicates all the other drives, which have a mixture of both environments. An illustration of the three scenarios are provided in Figure 2. This classification is done to compare the absolute and relative performance of the RTKLIB with respect to the baseline solution.

**Figure 3:**
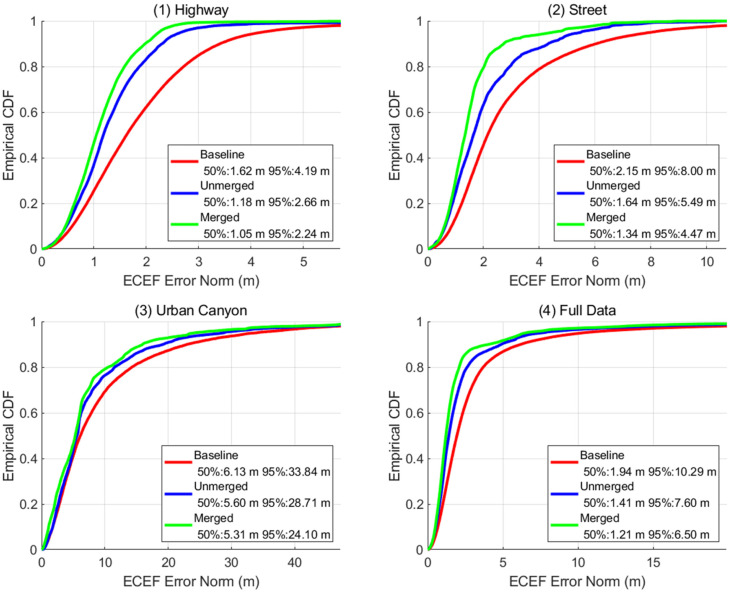
CDF plots of the performance of baseline solutions, unmerged RTKLIB solutions, and final merged solutions for each scenario and the entire training dataset: (**1**) Highway, (**2**) Street, (**3**) Urban Canyon, (**4**) Full Data. The unmerged RTKLIB solution was better than the baseline positions in all scenarios, and the merge further improved the accuracy of the RTKLIB solution.

For the accuracy assessment, GSDC requires only the latitude and longitude coordinates. Therefore, in the training set assessment, the altitude for the solutions was set to be the same as the truth. This effectively allows us to attain only the horizontal error. When we look at the cumulative distribution function (CDF) for all the scenarios, as shown in Figure 3, it is clear that the RTKLIB solution was more accurate than Google’s provided baseline solution in all scenarios. In addition, by merging the RTKLIB solutions, the accuracy of the final positions improved further. Overall, the baseline solution had 1.94 m for 50th percentile and 10.29 m for the 95th percentile, resulting in a score of 6.11. On the other hand, the merged RTKLIB solution attained 1.21 m for the 50th percentile and 6.5 m for 95th percentile, resulting in a score of 3.86. In terms of relative performance improvements, RTKLIB was the most effective over the baseline for the highway scenario. However, even for the absolute metrics, the unmerged RTKLIB provided almost 0.5 m better 50th percentile and 1.5–5.1 m better 90th percentile performance than the baseline. Furthermore, a limitation exposed by this CDF is that although RTKLIB is effective for open-sky conditions, the difficulty of mitigating multipath residuals remains.

**Figure 4:**
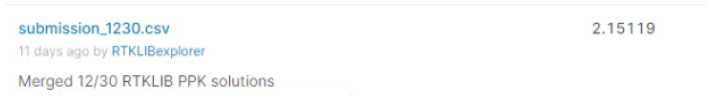
Screenshot of the official Kaggle private leaderboard results for the proposed RTKLIB PPK solution.

In order to see how the RTKLIB solution compares to the other participants, the test set results were uploaded to the official Kaggle website. Although the competition is over, the Kaggle site can still be used to assess the test data. For the test data, based on the private leaderboard score, Google’s baseline solution scored 5.42 for Kaggle, which is in 692th place out of 810 participants, while the proposed RTKLIB PPK solution scored 2.15 m, which resulted in 5th place, as shown in Figure 4. The private leaderboard score is provided instead of the public leaderboard counterpart, because different subsets of data are used for each scoring scheme, and the private score is used to determine the winners of the contest.

The main differences between the baseline solutions that Google provided as an example and the proposed solutions are how the GNSS measurements are computed and how the computed position solutions are processed [10]. For the navigation engine, the Google baseline solution used all satellite constellations, including GPS, GLONASS, BeiDou, Galileo, and QZSS, while the RTKLIB used GPS, GLONASS, and Galileo. For the raw measurements, the baseline solution used only pseudorange, but the RTKLIB also used carrier phase measurements for greater measurement precision. For the measurement processing, the baseline solution used the Klobuchar ionosphere model, EGNOS troposphere model, and ephemeris satellite clock model for error mitigation in single point positioning (SPP), while RTKLIB used double-differencing to remove atmospheric, clock, and orbital errors in its PPK solution. Moreover, while the baseline solution used weighted least squares for data processing, a Kalman filter was used for the RTKLIB in order to alleviate the impacts of measurement noise on the position solution. For the position solution processing, instead of providing the position solution from each device directly, the solutions were merged between devices based on the expected accuracies of the navigation solutions. This is done to mitigate the sections of data where carrier phase measurements from the phones are unreliable or unavailable. This process is difficult for the baseline solution, as no metrics are provided for the expected accuracy of the computed GNSS positions, but in the case of RTKLIB, estimates for the accuracy are provided by the navigation engine.

## 4. Summary and Conclusions

The latest version of the RTKLIB code, with the aforementioned changes and settings, was able to obtain 5th place ranking, with a score of 2.15. This is a significant improvement compared to the Google baseline score of 5.42, which implies that for the individuals using RTKLIB as the GNSS analysis tool, the provided tools, strategies, and updated code base provide an improved baseline from which to work. The code for this analysis used doppler-carrier comparison, geometry-free linear combination, and outlier detection methods for cycle slip detection, but did not incorporate any other cycle slip detection or mitigation methodologies, measurement error analysis, multipath mitigation, Kalman filter tuning, nor any of the numerous post-processing techniques proposed in various academic publications. Therefore, further performance improvements are expected with their additions. Furthermore, the conversion from the GnssLogger to RINEX can be further investigated, along with the optimization of the receiver parameter settings.

The improvement in the GNSS-derived positions compared to Google’s baseline solution is a crucial component of GSDC. This is because the competition has essentially two components: deriving accurate GNSS positions, and post-processing the GNSS solutions. The updated RTKLIB addresses the first component by proposing methods to improve the accuracy of the GNSS solutions and provide the resulting positions. This opens the door to more possibilities in the second component. For those who use Google’s baseline solution for post-processing, the RTKLIB GNSS positions will have the potential to provide results with higher accuracy. One of the strategies for the post-processing of the GNSS positions would be map matching with respect to the truth locations of the train data, as the drive trajectories on the road would be similar between collections.

All of the updates for the special GSDC version of RTKLIB are currently in the mainstream demo5 code in the latest b34f release. Moreover, a subset of the demo5 RTKLIB PPK solution code translated to python is available as a base for users who are interested in performing more involved experiments that can be difficult to develop in the original C code environment. Detailed steps required to replicate the results provided in this paper are provided in the blog [22].

## Figures and Tables

**Table 1:** Summary of changes to the demo5 b34eRTKlib code default settings file f9p_ppk.conf.

Positioning mode: static -> kinematic
GNSS constellations: disable BeiDou
Enable L5/E5 frequency: L1 + L2/E5b -> L1 + L2/E5b + L5/E5a
Filter Type: Combined -> Combined—no phase reset
SNR Mask L1/L5 (dB-Hz): 35/0 -> 24/24
Integer Ambiguity Res: on -> off
Cycle Slip Threshold: Geometry-Free: 0.05 -> 0.10
Cycle Slip Threshold: Doppler: N/A -> 5.0
Innov (m): 2 -> 1
Time Format: hms -> tow
Phase Error Ratio L5: 300 ->100
Carrier Phase Bias: 0.0001 -> 0.01 cycles

**Table 2:** Scenario and device combinations for the datasets that were excluded due to the presence of hardware clock discontinuities.

Scenario–Device	Total Epochs	Total Discontinuities
2020-05-14-US-MTV-2_Pixel4XLModded	129	33
2020-09-04-US-SF-1_Pixel4	258	253
2021-01-04-US-RWC-1_Pixel4Modded	263	222
2021-01-04-US-RWC-1_Pixel4XL	272	272
2021-01-04-US-RWC-2_Pixel4Modded	262	262
2021-01-04-US-RWC-2_Pixel4XL	266	266
2021-01-05-US-SVL-1_Pixel4XL	263	222
2021-01-05-US-SVL-1_Pixel5	269	268
2021-01-05-US-SVL-2_Pixel4XL	257	197
2021-03-10-US-SVL-1_Pixel4XL	242	242
2021-04-29-US-MTV-1_Pixel4	235	121
2021-04-29-US-MTV-1_Pixel5	261	249

## Data Availability

The GSDC data can be found on the official Kaggle website: https://www.kaggle.com/c/google-smartphone-decimeter-challenge/data, accessed on 13 April 2022. The observation data for the nearby CORS station can be found at NOAA: https://www.ngs.noaa.gov/UFCORS/, accessed on 13 April 2022. The BRDM files for the satellites can be found at IGS: https://igs.bkg.bund.de/root_ftp/IGS/BRDC/, accessed on 13 April 2022. The original RTKLIB code is found on the official website: http://www.rtklib.com/, accessed on 13 April 2022. The GnssLogger to RINEX conversion script and the updated RTKLIB code can be found on GITHUB: https://github.com/rtklibexplorer/RTKLIB/releases/tag/b34e_smartphone, accessed on 13 April 2022. The python subset of RTKLIB code for PPK solutions can be found at: https://github.com/rtklibexplorer/rtklib-py, accessed on 13 April 2022.
